# Applicability of American College of Radiology Appropriateness Criteria Decision-Making Model for Acute Appendicitis Diagnosis in Children

**DOI:** 10.3390/diagnostics12122915

**Published:** 2022-11-23

**Authors:** Ozum Tuncyurek, Koray Kadam, Berna Uzun, Dilber Uzun Ozsahin

**Affiliations:** 1 Kolan British Hospital, TRNC Mersin 10, Nicosia 99138, Turkey; 2 Department of Radiology, Cyprus International University, TRNC Mersin 10, Nicosia 99138, Turkey; 3 Department of Emergency, Faculty of Medicine, Near East University, TRNC Mersin 10, Nicosia 99138, Turkey; 4 Department of Statistics, Carlos III de Madrid University, 28903 Madrid, Spain; 5 Department of Mathematics, Faculty of Arts and Sciences, Near East University, TRNC Mersin 10, Nicosia 99138, Turkey; 6 Medical Diagnostic Imaging Department, College of Health Science, University of Sharjah, Sharjah 27272, United Arab Emirates; 7 Operational Research Center in Healthcare, Near East University, TRNC Mersin 10, Nicosia 99138, Turkey

**Keywords:** appendicitis, diagnosis, fuzzy logic, decision making

## Abstract

Acute appendicitis is one of the most common causes of abdominal pain in the emergency department and the most common surgical emergency reason for children younger than 15 years of age, which could be enormously dangerous when ruptured. The choice of radiological approach is very important for the diagnosis. In this way, unnecessary surgery is avoided. The aim of this study was to examine the validity of the American College of Radiology appropriateness criteria for radiological imaging in diagnosing acute appendicitis with multivariate decision criteria. In our study, pediatric patients who presented to the emergency department with abdominal pain were grouped according to the Appendicitis Inflammatory Response (AIR) score and the choice of radiological examinations was evaluated with fuzzy-based Preference Ranking Organization Method for Enrichment Evaluation (PROMETHEE) and with the fuzzy-based Technique for Order of Preference by Similarity to Ideal Solution (TOPSIS) model for the validation of the results. As a result of this study, non-contrast computed tomography (CT) was recommended as the first choice for patients with low AIR score $({\mathrm{where}\;\Phi }^{\mathrm{net}}=0.0733$) and with high AIR scores (${\mathrm{where}\;\Phi }^{\mathrm{net}}=0.0702$) while ultrasound (US) examination was ranked third in patients with high scores. While computed tomography is at the forefront with many criteria used in the study, it is still a remarkable practice that US examination is in the first place in daily routine. Even though there are studies showing the strengths of these tools, this study is unique in that it provides analytical ranking results for this complex decision-making issue and shows the strengths and weaknesses of each alternative for different scenarios, even considering vague information for the acute appendicitis diagnosis in children for different scenarios.

## 1. Introduction

Acute appendicitis is the most common indication reason requiring emergency abdominal surgery [1,2,3]. Acute appendicitis (AA) is the most common surgical emergency in younger than 15 years old children [4]. Surgical treatment is the first option for this diagnosis. Although the treatment options are improved, there are 510 morbidity and 1–5% mortality rates, while there is a 5–42% negative appendectomy rate [2,3,5]. This causes 3 billion dollars of annual hospital spending according to USA data. Many biomarkers such as blood or urine analysis have been defined for diagnosis, but they are not definitive methods [6,7]. It can benefit from ultrasound, CT, and MRI in the diagnosis of appendicitis. Diagnosing acute appendicitis (AA) in children remains challenging for physicians due to atypical presentations [8,9,10]. Proper diagnosis of AA can prevent complications such as perforation and abscess [11]. Ultrasound is an imaging technique with high specificity in diagnosis in the pediatric group. However, it is operator-dependent [12]. However, CT, which has higher specificity, has a narrow area of use due to X-ray exposure [13] and MRI for claustrophobia. US scanning was used initially, but CT scanning has become increasingly used as a diagnostic tool in adults as well as children to make a more accurate diagnosis and rule out appendicitis [14]. However, it is a controversial issue, as it is known that exposure to CT scanning increases the risk of cancer in the long term, especially in children [15]. There are many scoring systems for appropriate diagnostic techniques and surgical decisions in the management of right lower quadrant pain in pediatric patients in the emergency department [16]. The Appendicitis Inflammatory Response (AIR) score is AUROC 0.96 in the widest age range and enables us to avoid CT application [17]. The AIR score is one of the two scores (the other one is ‘Adult Appendicitis Score’, AAS) recommended by the 2020 World Society of Emergency Surgery clinical practice guidelines for the diagnosis and treatment of acute appendicitis [18]. The parameters of the AIR score are vomiting, right lower quadrant (RLQ) pain, rebound tenderness, body temperature, polymorphonuclear leukocytes (PNL), WBC count, and C-reactive protein (CRP) level [19] (Table 1). However, literature information on the applicability of these scores and their compatibility with the ACR eligibility criteria in the field is limited.

Many multivariate decision criteria have been developed in medical studies for many machine learning algorithms and approaches. For diagnostic and classification purposes, conceptual decision-making models are most often used. Decision trees provide a useful and effective decision-making technique with high classification precision and these models are easy to compare to most classification algorithms. For the diagnosis of acute appendicitis, the sensitivity and specificity values of ultrasound are 78% and 83%, respectively [18], 100% and 94.8% for contrast-enhanced CT, and 90.5% and 100% for non-contrast CT, respectively [19]. The sensitivity and specificity values for 1.5 T MRI are 96.6% and 95.8%, respectively [20], and 100% and 98% for 3T MRI, respectively. Tseng et al. [21] showed that ultrasound was the cause of the highest rate of negative appendectomy. When CT imaging was added, there was a 0.6% reduction. Data from the National Surgical Quality Improvement Program (NSQIP) were used in this recent study. We wanted to test the ACR eligibility criteria with a new model that has not been studied in the literature before, in order to minimize the confusion in the diagnostic process. The purpose of this study is to determine which examination is the most appropriate for patients with low/high AIR score in the diagnosis of acute appendicitis.

Table 1 displays the score of the appendicitis inflammatory response for various diagnosis occurrence conditions.

In order to achieve our aim, we applied multi-criteria decision-making analysis approaches, specifically the fuzzy-based Preference Ranking Organization Method for Enrichment Evaluation (PROMETHEE) and the fuzzy-based Technique for Order of Preference by Similarity to an Ideal Solution (TOPSIS), to discover the strengths and weaknesses of each diagnosis technique for the acute appendicitis diagnosis in children using the multi-parameters assigned with the alternative’s medical tools for the diagnosis.

## 2. Material and Method

### 2.1. Study Design

After Institutional Review Board approval was obtained (YDU/2020/85-1186), data of all cases under 16 years of age admitted 2019–2020 with diagnosis of the acute abdominal pain were collected. The diagnosis of the patients was confirmed by two researchers through analysis of all records of the patients. All patients were free of tumor, infection, and hematological diseases. As data for the analysis, the examinations were assessed by two expert observers (O.T., K.K.) in terms of imaging time, easy applicability of the examination, duration of the examination, specificity, sensitivity, user dependency, fee, diagnostic precision, request duration, and radiation exposure, and with their joint decision. Their answers were defined as five scale linguistic triangular fuzzy scales. The model made an examination selection ranking according to the AIR score data. This analysis was made for US, non-contrast CT, contrast-enhanced CT, 1.5 T MRI, and 3T MRI. Furthermore, the PROMETHEE and the TOPSIS techniques were applied for this evaluation. The Gaussian preference function was used for the PROMETHEE analysis for each criterion. Linguistic fuzzy scale was applied for the determination of the importance weights of the criteria and selected criteria and Yager index was used for the defuzzification of the defined triangular fuzzy values.

**Figure 1:**
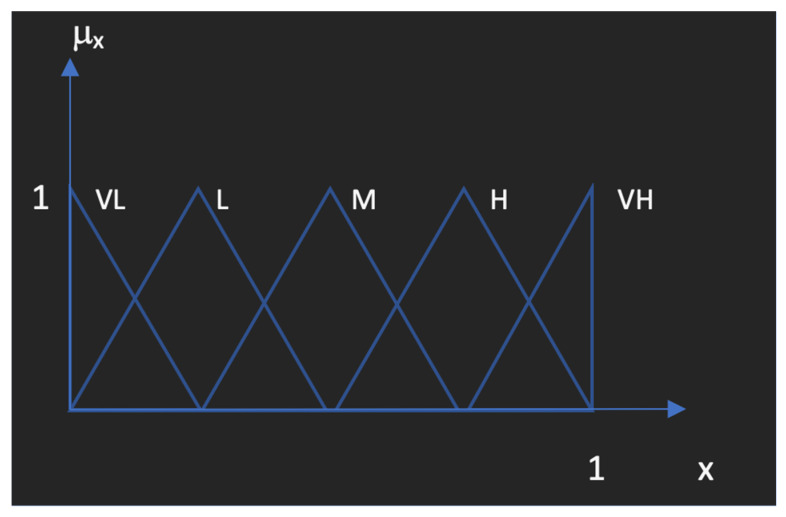
Linguistic fuzzy sets.

Figure 1 shows the linguistic fuzzy sets and their assigned fuzzy numbers used for the expression of the fuzzy parameters. VH: Very high; H: High; M: Medium; L: Low; and VL: Very low are defined as the linguistic expressions used for determination of the data. Each linguistic expression and the importance given to parameters selected by experts are assigned to triangular fuzzy values as shown in detail at Table 2 below.

Using the fuzzy scale enables the expert to determine the weights or degrees of importance of the parameters used for the comparison of the medical diagnosis tools for acute appendicitis in pediatric patients. Then, after the defuzzification process, the decision matrix is constructed for use in MCDM approaches. Yager index is a value that provides the defuzzified point for the triangular fuzzy numbers, proposed by Ronald Yager as one of the most important ranking methods for fuzzy sets.

Data: Data included demographics, preoperative clinical findings for AIR score such as vomiting, pain in RLQ, rebound tenderness or muscular defense, body temperature, polymorphonuclear leukocytes rate, white blood cell count, and C-reactive protein concentration. Radiological data were not included in generator operator-independent data. Parental consent was obtained both of surgical approach and publication of the data. Diagnosis and management of appendicitis were determined through clinical, laboratory, radiological, and surgical findings.

Statistical analysis: Statistical analysis was performed with IBM SPSS Statistics 26.0.0 (Chicago, IL, USA). The characteristics of the study sample were analyzed by descriptive statistics, with dichotomous or ordinal data presented as percentage, and continuous data as means with SD. The Kolmogorov–Smirnov test was used to demonstrate normal distribution. One-way ANOVA was used for homogeneity of variables, while Student’s *t* test was used. Statistical associations were considered significant if the *p* value was <0.05. In our daily life, we face many problems in different fields, the most common ones are the problems related to choosing or evaluating something included in groups of other choices. In some cases, the evaluation of the situations could be complex and needs to consider many factors with different weights or levels of importance for these factors for the evaluation. To solve this problem, multi criteria decision making (MCDM) is used and this principle refers to making decisions in the presence of multiple, usually conflicting, criteria [21]. Although MCDM problems are very common and popular, MCDM as a discipline has a short history of about 30 years. It developed relatively to computer advancements and development and because of this relation, computers gave us the ability to conduct complex and big MCDM problems which expands the applications of MCDM.

On the other hand, the popularity of computers and mobiles generated an enormous amount of data in several fields which makes MCDM more important and dominant in supporting design makers in different sectors with usable data [21]. The MCDM problems have three main components which are the decision maker/s (DMs), alternatives, and criteria. In general, the classification of MCDA problems depends on types of these three elements. Decision maker/s (DMs): in problems, we may have one decision maker which is responsible for determining what to do or multiple decision makers such as several people or organizations who are involved in the processes of MCDM. In case of multiple decision making, many different preferences, goals, criteria, and objectives appear so the results might not satisfy every DM [22]. In this case, obtaining usable outcomes depends mainly on the range of cooperation between the DMs. In case of the presence of multiple DMs with different preferences and priorities, the problem of their presence could be included in MCDM problems to be solved [23]. Alternatives: these are the things or possibilities that the decision maker/s should choose from. These possibilities could be identified previously or could be created through process. It is important to know that the decision space is the definition of the set of all possible alternatives [22]. Criteria: the specifications or requirements that each element of decision space should have or possess and depending on these requirements, each element is rated and evaluated by how well it possesses one of the process criteria [22].

There are many ways to classify the methods of multi criteria decision making. One of these ways is to classify according to decision makers which could be one decision maker or group of decision makers; the methods could also be classified according to the type of data used in the process, such as deterministic data which are accurate and clear data, stochastic which means the data are random and the opposite of deterministic data, and in some cases the data presented as fuzzy information which are not clear or vague. There are some subsidiary classifications such as in case of a deterministic, single decision maker. The classification problem considers the type of information and the number of decision makers [24]. Multi criteria decision making has many techniques such as Analytic Hierarchy Process (AHP) method, Preference Ranking Organization Method for Enrichment Evaluation (PROMETHEE), Technique for Order of Preference by Similarity to Ideal Solution (TOPSIS), Analytical Network Process (ANP), Compromise ranking method (VIKOR), Elimination Et Choix Traduisant la Realité (ELECTRE) [25,26], etc. In this study, two types of analytical methods were used, specifically F-PROMETHEE and F-TOPSIS.

### 2.2. Fuzzy PROMETHEE (F-PROMETHEE)

This definition or technique contains two main parts, fuzzy and PROMETHEE. With the fuzzy logic process, the vague and linguistic data could be defined and with the PROMETHEE process, the ranking of the alternatives under the fuzzy conditions can be obtained. The PROMETHEE method was created by Brans et al. (1984) in order to present a rational and clear method to rank alternatives [27,28]. The first part of the technique is the fuzzy process, which refers to mathematical means used to explain the non-numerical data or uncertain information mathematically. Distinguishing the real value depends on if it is completely true or false. This manner facilitates the utilizing, interpreting, and manipulating the data when there is a lack of certainty or clearance [29]. The second part is PROMETHEE and this method is used for evaluating and arranging the alternatives with different criteria to get a specific aim. This technique has two parts, PROMETHEE I and II; the first one used for obtaining the partial raking results and the second one used to get the net ranking results between the alternatives [9]. PROMETHEE is one of the outranking methods, which is well understood and has a lot of applications in many fields. There are mainly six steps of this method as shown below [30,31] after the decision matrix, the matrix that contains alternatives and the criterion, is constructed:

Step 1. Determination of the preference function for each criterion $\left(p_{k}\right)$.

Step 2. Determination of the importance weights for each criterion ($w_{k}$).

Step 3. Calculation of the outranking relation/preference index $\pi \left(.\right)$ for each pair of alternatives with the Equation (1):

(1) $$\pi \left(a_{t},a_{t^{\prime }}\right)=\overset{K}{\underset{k=1}{\sum }}w_{k}.\left[p_{k}\left(f_{k}\left(a_{t}\right)-f_{k}\left(a_{t^{\prime }}\right)\right)\right],\;\;AXA\to \left[0,\;1\right]$$

$\pi \left(a_{t},a_{t^{\prime }}\right)$ stands for the preference index of the alternative $a_{t}$ compared to alternative $a_{t^{\prime }}$ by considering each criterion simultaneously and $f_{k}\left(a_{i}\right)$ denotes the value of the *k*-th criterion of *i*-th alternative. 

Step 4. Calculations of the entering/negative outranking flow and leaving/positive outranking flow for each alternative using the Equations (2) and (3), respectively.

(2) $$\Phi^{-}\left(a_{t}\right)=\frac{1}{n-1}\overset{n}{\underset{t^{\prime }=1\;t^{\prime }\neq t\;}{\sum }}\pi \left(a_{t^{\prime }},a_{t}\right)$$

(3) $$\Phi^{+}\left(a_{t}\right)=\frac{1}{n-1}\overset{n}{\underset{t^{\prime }=1\;t^{\prime }\neq t\;}{\sum }}\pi \left(a_{t},a_{t^{\prime }}\right)$$

where *n* denotes the number of alternatives.

The leaving flow indicates how good the alternative $a_{t}$ is over other alternatives, while the entering flow indicates how much all other alternatives are better or more preferred than alternative $a_{t}$ and it shows how weak the alternative $a_{t}$ is.

Step 5. Generating the partial order of the alternatives based on the following statements:

$a_{t}$ should be preferred to $a_{t^{\prime }}$ ($a_{t}Pa_{t^{\prime }})$ if

(4) $$\{\Phi^{+}\left(a_{t}\right)>\Phi^{+}\left(a_{t^{\prime }}\right)\;and\;\Phi^{-}\left(a_{t}\right)\leq \Phi^{-}\left(a_{t^{\prime }}\right)\;\Phi^{+}\left(a_{t}\right)=\Phi^{+}\left(a_{t^{\prime }}\right)\;and\;\Phi^{-}\left(a_{t}\right)<\Phi^{-}\left(a_{t^{\prime }}\right)$$

$a_{t}$ is equally preferred to $a_{t^{\prime }}$ ($a_{t}Ia_{t^{\prime }})$ if

(5) $$\left(a_{t}Ia_{t^{\prime }}\right)\mathrm{if}:\;\Phi^{+}\left(a_{t}\right)=\Phi^{+}\left(a_{t^{\prime }}\right)\;\;and\;\Phi^{-}\left(a_{t}\right)=\Phi^{-}\left(a_{t^{\prime }}\right)$$

$a_{t}$ is incomparable to $a_{t^{\prime }}(a_{t}Ra_{t^{\prime }}$) if

(6) $$\{\Phi^{+}\left(a_{t}\right)>\Phi^{+}\left(a_{t^{\prime }}\right)\;and\;\Phi^{-}\left(a_{t}\right)>\Phi^{-}\left(a_{t^{\prime }}\right)\;\Phi^{+}\left(a_{t}\right)<\Phi^{+}\left(a_{t^{\prime }}\right)\;\;\;\;and\;\Phi^{-}\left(a_{t}\right)<\Phi^{-}\left(a_{t^{\prime }}\right)$$

The last statement occurring in this PROMETHEE I process the total ranking can be obtained by applying to PROMETHEE II method.

Step 6. The net ranking results should be determined by using Equation (7)

(7) $$\Phi^{net}\left(a_{t}\right)=\Phi^{+}\left(a_{t}\right)-\Phi^{-}\left(a_{t}\right)$$

Total ranking results can be obtained with the following statements.

(8) $$\left(a_{t}Pa_{t^{\prime }}\right)\;\mathrm{if}\;\Phi^{net}\left(a_{t}\right)>\Phi^{net}\left(a_{t^{\prime }}\right)$$

(9) $$\left(a_{t}Ia_{t^{\prime }}\right)\;\mathrm{if}\;\Phi^{net}\left(a_{t}\right)=\Phi^{net}\left(a_{t^{\prime }}\right)$$

The higher net flow ($\Phi^{net}\left(.\right)$ means a better alternative and that demonstrates the aim of PROMETHEE II (10).

### 2.3. TOPSIS

The TOPSIS method was created as an alternative to the ELECTRE method by Yoon and Hwang 1979 [11,32]. This method is based on the principle of when the alternative has the shortest distance to the ideal solution, that indicates that it is the best choice.

In the classical TOPSIS method, in the presence of one decision maker, the assumption is that the ratings and the weights are well represented by numerical data. The assumption differs and is more complex if there are many decision makers, because the preferences and vision differ. There are mainly 5 steps for the TOPSIS process as follows [12]:

Step 1: Creation of decision matrix $X={\left[x_{ij}\right]}_{mxn}$ and the importance weights ($w_{j}$) where

(10) $$\sum_{1}^{n}w_{j}=1$$

Step 2. Calculate the normalized decision matrix; the commonly used normalization data can be calculated by using the Equation (11):

(11) $$n_{ij}=\frac{X_{ij}}{\sqrt{\sum_{i=1}^{m}X_{ij}^{2}}}$$

Step 3. Calculate the weighted normalized decision values ($\;v_{ij}$) to obtain the weighted normalized matrix using the Equation (12).

(12) $$\;v_{ij}=w_{j}n_{ij}$$

where i=1,…,m;j=1,…,n.

Step 4. Determine the positive ideal solution (PIS/$A^{+}$) and negative ideal solution (NIS/$A^{-}$). In this step, two extreme sides (negative and positive) are identified as given in Equations (13) and (14).

(13) $$A^{+}=(v_{1}^{+},v_{2}^{+},\ldots ,v_{n}^{+})=\left[\left[v_{ij}\;|j\in I\right],\left[v_{ij}\;|j\in J\right]\right]$$

(14) $$A^{-}=(v_{1}^{-},v_{2}^{-},\ldots ,v_{n}^{-})=\left[\left[v_{ij}\;|j\in I\right],\left[v_{ij}\;|j\in J\right]\right]$$

where i=1,…,m;j=1,…,n, I denotes the beneficial criteria and J denotes the non-beneficial criteria.

Step 5. Calculate the distance measures from the positive ideal solution ($d_{i}^{+}$) and the negative ideal solution $\left(d_{i}^{-}\right)$ for each alternative by using the *n* dimensional Euclidean metric using the following equations:

(15) $$d_{i}^{+}=\sqrt{\sum_{j=1}^{n}{\left(v_{ij}-v_{j}^{+}\right)}^{2}},\;i=1,2,\ldots ,m$$

(16) $$d_{i}^{-}=\sqrt{\sum_{j=1}^{n}{\left(v_{ij}-v_{j}^{-}\right)}^{2}},\;i=1,2,\ldots ,m$$

Step 6. Rank the alternatives based on the relative closeness to the positive ideal solution ($R_{i}$) in descending order where:

(17) $$R_{i}=\frac{d_{i}^{-}}{d_{i}^{-}+d_{i}^{+}}$$

## 3. Results

Of 51 patients, who were admitted in 2019–2020 to the Emergency Department with abdominal pain, ultrasound (US) was performed in 96% (*n*: 49) patients. Computed tomography (CT) was performed in 0.7% (*n*: 4) patients. In 69% (*n*: 34) of the patients, the US result was acute appendicitis. In 31% (*n*: 15) patients, appendicitis was not detected on ultrasound. The mean AIR score of patients diagnosed with acute appendicitis by US (*n*: 34) was 6 ± 1.8 (mean ± SD), and the patients not diagnosed with appendicitis (*n*: 15) were 4.6 ± 2.3 (mean ± SD) (*p* = 0.03). The mean AIR score of patients diagnosed with acute appendicitis with CT was 7.5 ± 1.3 (mean ± SD), and the patients without CT were 5.5 ± 2 (mean ± SD) (*p* = 0.04). 72% (*n* = 37) of the patients had undergone surgery. The AIR score was 6.3 ± 1.6 (mean ± SD), and the 14 patients who did not undergo appendectomy were 3.9 ± 2.2 (mean ± SD) (*p* = 0.00). For the cost of 51 patients, it was an average of 752.3 ± 549 USD (mean ± SD), while the AIR score value of 52% (*n* = 27) patients costing more than 750 USD was 6.37 ± 1.6, the mean AIR score value of the remaining 24 patients was found to be 4.96 ± 2.3 (*p* = 0.01). The mean duration of hospital stay was 1.92 ± 2.1 days for 51 patients, while the mean AIR score value of 30 patients staying more than two days was 6.43 ± 1.7, and the mean AIR score value of 21 patients staying one day was 4.67 ± 2.1 (*p* = 0.00). In addition, four patients without any findings of appendicitis in the US were operated on. One of them was diagnosed with appendicitis on CT and the air score was 6. The other three patients were operated on due to an increase in AIR score (mean AIR score: 7) without additional cross-sectional imaging. In two patients, patients who did not accept surgery (mean AIR score: 5.5) even though the US diagnosis was appendicitis were discharged with medical treatment.

### Multi Criteria Decision Making Theory Results (MCDM)

If the patient’s AIR score is below 6, non-contrast CT $\left(\Phi^{net}=0.0733\right)$ should be selected as the first display option. The second option was contrast enhanced CT $\left(\Phi^{net}=0.0535\right)$, then US $\left(\Phi^{net}=0.1305\right)$ and MRI $\left(\Phi^{net}=-0.0432\right),$ in that order, based on the fuzzy-PROMETHEE ranking result as displayed in Table 3.

**Figure 2:**
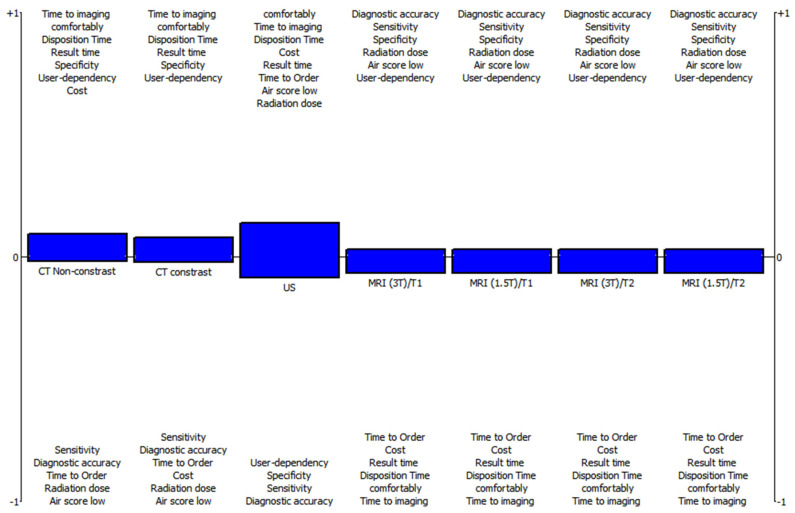
Evaluation of the acute appendicitis diagnosis for low AIR score with F-PROMETHEE.

The graphic in Figure 2 shows which test to choose for the patient group with low AIR score based on the F-PROMETHEE approach.

The detailed ranking results by the F-TOPSIS method are shown in Table 4, where the patients’ air score is low. Using the F-TOPSIS technique, we obtained the ranking results, where the air score is low, as the best option is CT-non-contrast ($R_{i}=0.6315$), and the last option is MRI ($R_{i}=0.5217$) as similar to F-PROMETHEE results.

If the patient’s AIR score is above 6, F-PROMETHEE results shows that non-contrast CT $\left(\Phi^{net}=0.0702\right)$ should be chosen as the first imaging option. The second option was US $\left(\Phi^{net}=0.0617\right)$, contrast-enhanced CT $\left(\Phi^{net}=0.0503\right)$, or MRI $\left(\Phi^{net}=0.0456\right)$, in that order, as displayed in Table 5.

**Figure 3:**
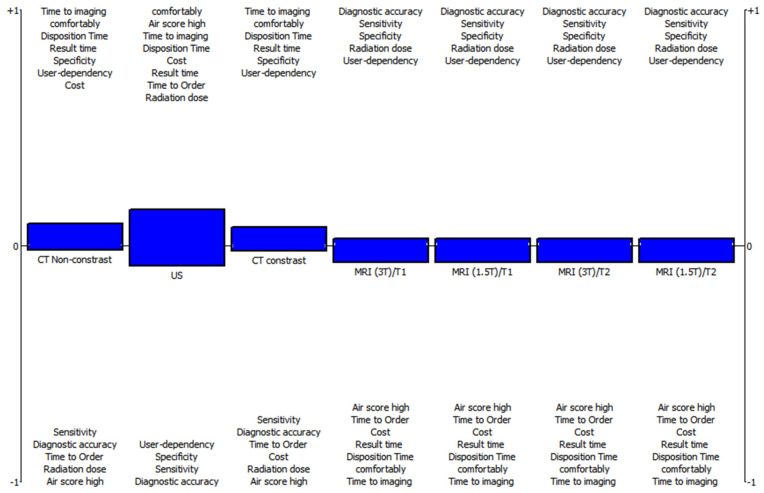
Evaluation of the acute appendicitis diagnosis for high AIR score patients with F-PROMETHEE.

The graphic in Figure 3 shows which test to choose for the patient group with high AIR score based on the F-PROMETHEE approach.

As seen in Table 6. using the F-TOPSIS technique, we obtained the ranking results, where the air score is high, as the best option is CT-non-contrast ($R_{i}=0.6191$), and the last option is MRI ($R_{i}=0.5153$) as similar to the F-PROMETHEE results.

## 4. Discussion

The result we found in the appendicitis diagnosis algorithm matches the pediatric patient group of our sample group. With this study, mathematical modeling was used for the first time in the literature for the diagnosis of acute appendicitis in pediatric patients. There are no similar studies in the literature. The method is a support system with which imaging will be more appropriate when pediatric patients suffer from appendicitis. In this study, a radiology and an emergency medicine specialist entered the MCDM program in line with the ACR appropriateness criteria. Consistent with the result in the study group, if ultrasound is preferred in the group with low AIR score, treatment can be performed quickly. The use of a cross-sectional imaging method, CT or MRI, is seen as a method that will facilitate diagnosis and treatment in patients with high AIR scores for whom US examination may be insufficient. This model is a decision support system that allows for which examination to choose for diagnosis in the emergency department. In this model, the weight of importance can be updated according to the expert doctor. According to the literature update, the criteria for this decision support system can be expanded, new imaging technologies can be added and easily renewed.

The criteria chosen in our study were imaging time, easy applicability of the examination, duration of the examination, time to result, specificity, sensitivity, user dependency, cost, diagnostic precision, request time, and radiation exposure level. Benabbas [4] et al. showed that while testing the superiority of ED point-of-care US over MR and CT, they did not use such factors as treatment cost, applicability of imaging method, and length of hospital stay, but we also included these factors in our study. Results depend on the observer. The advantage of this is that it enables experienced people to share their clinical practice approach. Thus, data can be globalized. Local differences are important. However, it is easy to update the program for these as well.

Aydın et al. [33] used blood tests decision trees for acute appendicitis diagnosis. We compared imaging techniques for diagnosis. With a similar thought, we tested which of the imaging methods used for diagnosis was more helpful in our study.

We did not use only one model in our study. We proved the accuracy of the results with the second model. In this way, we had an objective approach. It has been observed that the criteria of MRI devices for abdominal imaging are identical. Therefore, no difference was observed in the order of these devices. The first limitation of the study is the limited number of observers. We aimed to convey the experiences of both the emergency service and the radiology specialist in our own sample group. The second limitation is the retrospective analysis of the sample group. In conclusion, while computed tomography is at the forefront with many criteria used in the study, it is still a remarkable practice that US examination is in the first place in daily routine.

In conclusion, CT is the most reliable method for diagnosis with our model. Although it contains radiation that reduces the use of this examination, it is superior to the US examination in daily practice in order to minimize the damage that the patients may experience during the treatment process.

## Figures and Tables

**Table 1:** Appendicitis Inflammatory Response (AIR) score.

Diagnosis	Score
Vomit	1
Pain in RLQ	1
Rebound tenderness low	1
Rebound tenderness mild	2
Rebound tenderness severe	3
Temperature > 38.5 °C	1
PNL 70–84%	1
PNL > 85%	2
Leukocytes (WBC) > 10.0–14.9 × 109/L	1
Leukocytes (WBC) > 15.0 × 109/L	2
CRP 10–49 g/L	1
CRP > 50 g/L	2

AIR: if sum 0–4 = low probability; if sum 5–8 = mild probability; if sum 9–12 = high probability.

**Table 2:** Linguistic fuzzy scale.

Linguistic Scale for Evaluation	Triangular Fuzzy Scale	Criteria of the Decision Alternatives
VH	(0.75, 1, 1)	specificity, diagnostic precision, time of imaging, user dependency, radiation dose, comfortability
H	(0.50, 0.75, 1)	sensitivity, air score, disposition
M	(0.25, 0.50, 0.75)	time of order, result time, cost
L	(0, 0.25, 0.50)	-
VL	(0, 0, 0.25)	-

VH: Very high; H: High; M: Medium; L: Low; VL: Very low.

**Table 3:** Ranking of the acute appendicitis diagnosis for low AIR score by F-PROMETHEE approach.

Rank	Alternatives	$\Phi^{net}$
1	CT non-contrast	0.0733
2	CT contrast	0.0535
3	US	0.1305
4	MRI	−0.0432

**Table 4:** Ranking of the acute appendicitis diagnosis for low AIR score by F-TOPSIS approach.

Rank	Alternatives	$d_{i}^{+}$	$d_{i}^{-}$	*R_i_*
1	CT non-contrast	0.0927	0.1588	0.6315
2	CT contrast	0.0946	0.1561	0.6227
3	US	0.1210	0.1435	0.5425
4	MRI	0.1288	0.1405	0.5217

**Table 5:** Ranking of the acute appendicitis diagnosis for high AIR score by F-PROMETHEE approach.

Rank	Alternatives	$\Phi^{net}$
1	CT non-contrast	0.0702
2	US	0.0617
3	CT contrast	0.0503
4	MRI	0.0456

**Table 6:** Ranking of the acute appendicitis diagnosis for high AIR score by F-TOPSIS approach.

Ranking	Alternatives	$d_{i}^{+}$	$d_{i}^{-}$	*R_i_*
1	CT non-contrast	0.0977	0.1588	0.6191
2	CT contrast	0.0995	0.1561	0.6107
3	US	0.1210	0.1468	0.5481
4	MRI	0.1316	0.1399	0.5153

## Data Availability

The data used to support the findings of this study can be provided upon the request from the authors.
